# Work-life balance amongst dental professionals during the COVID-19 pandemic—A structural equation modelling approach

**DOI:** 10.1371/journal.pone.0256663

**Published:** 2021-08-24

**Authors:** Swathi Pai, Vathsala Patil, Rajashree Kamath, Mansi Mahendra, Deepak Kumar Singhal, Vishal Bhat

**Affiliations:** 1 Department of Conservative Dentistry and Endodontics, Manipal College of Dental Sciences, Manipal, Manipal Academy of Higher Education, Manipal, Karnataka, India; 2 Department of Oral Medicine and Radiology, Manipal College of Dental Sciences, Manipal, Manipal Academy of Higher Education, Manipal, Karnataka, India; 3 Economics and Qualitative Techniques, School of Business and Management, CHRIST (Deemed to be University), Bengaluru, Karnataka, India; 4 Manipal College of Dental Sciences, Manipal, Manipal Academy of Higher Education, Manipal, Karnataka, India; 5 Department of Public Health Dentistry, Manipal College of Dental Sciences, Manipal, Manipal Academy of Higher Education, Manipal, Karnataka, India; 6 Department of Pharmacology, Melaka Manipal Medical College, Manipal, Manipal Academy of Higher Education, Manipal, Karnataka, India; Post Graduate Institute of Medical Education and Research, INDIA

## Abstract

**Background:**

The Coronavirus disease (COVID-19) outbreak in 2019, has shocked the entire world. As an effort to control the disease spread, the Indian government declared a nationwide lockdown on March 25th, 2020. As dental treatment was considered high risk in the spread of COVID-19, dentistry became one of the most vulnerable professions during this time. Dental professionals had to face job layoffs, salary cuts in professional colleges, closure of private clinics resulting in huge psychological, moral, and financial crises. Studies during the previous and present pandemics have shown mental issues among health care workers necessitating institutional reforms, along with early care and support. A balance in the work-life amongst professionals is the key to better efficiency and, was majorly affected during the COVID-19 pandemic lockdown due to sudden unexpected changes. Hence this study was conducted to understand the changes they underwent both at home and professional front with a hypothesis that physical and mental health, activities, relationship status, and workplace influence the work-life balance.

**Methods:**

A pre-validated questionnaire survey was done on dentists across India. Structural Equation Modelling and path analysis were applied to the data collected.

**Results:**

The results of the study supported the hypothesis that factors like physical and mental health, activities, relationship status, and workplace influenced the work-life balance directly. A significant imbalance was seen amongst the female dentists.

**Conclusion:**

The present study proved the unpreparedness among dental professionals. Hence an evolutionary phase in every field with better working protocols, robust mental health support, and a focus on strategies to face future such emergencies is required.

## Introduction

By the end of year 2019, world got into the grip of a deadly novel Coronavirus and soon the outbreak was declared a public health emergency of international concern by World Health Organisation(WHO) [1]. Given the large population of over 1.3 billion, the Indian government declared a nationwide lockdown on March 25^th,^ 2020 as a part of its efforts to control the disease spread [2, 3]. All the regular outpatient departments across hospitals and clinics in India were closed and only the emergency healthcare services were available. Consequently, dental professionals across the country temporarily ceased performing clinical services, and only emergency treatments were performed using full Personal Protective Equipment (PPE) protection.

Due to the COVID-19 lockdown, there were job layoffs, salary cuts in professional colleges, private clinics and dentists were facing huge psychological, moral, and financial crises [3]. According to a study by Consolo U et al. (2020), approximately 85 percent of dentists in a district in Italy reported being worried about acquiring the infection during dental procedures [4]. Another study by Shacham M et al. (2020) on dental professionals in Israel showcased elevated psychological distress among dentists who had background illness, who feared contracting COVID-19 from patients, and who also had higher subjective overload [5]. However, lower psychological distress amongst those in a committed relationship and having higher self-efficacy was also noted [5].

As virtual training of dental skills is not yet well established in all the places of India, the closure of dental colleges also impacted dental training. The respiratory pandemic also provoked that standard protective measures in daily clinical work may be ineffective to prevent the spread of COVID-19, especially when patients are in the incubation period or as asymptomatic carriers. Thus, dentistry became one of the most vulnerable professions during the times of the COVID-19 Pandemic [3, 5].

A balance in work-life involves engagement in work and non-work life with a minimal conflict between the two roles [6]. A good work-life balance leads to high organizational performance, increased job satisfaction, and stronger organizational commitment [7]. It also plays an important role in individuals’ health, family, and overall satisfaction [8]. Previous pandemic outbreaks suggested that Health care workers (HCW) suffered psychological distress [9, 10]. Analysis during the present pandemic also has shown mental issues among health care workers necessitating early care and support [11]. Although Indian dentists showed satisfactory knowledge and preparedness for the COVID-19 pandemic, the fall in the economy and recession has led to rising anxiety, nervousness, fear of losing the job, frustration, and mental stress among the dentists [12, 13]. The dental profession has encountered multiple challenges like change in the cost of treatment, requirements of newer equipment, fear of taking new patients, patients canceling appointments due to fear and vulnerability to infection. Along with these professional challenges, changes in the house set up with people taking work from home, home-schooling for children, non-socializing, unable to go out, etc. have caused an imbalance in the work-life atmosphere [4].

Despite the availability of a large amount of epidemiological data on communicable diseases, there is a scarcity of knowledge and awareness about the psychological morbidities these pandemics cause on HCWs and especially the dental team [10]. Although, previous studies during the COVID-19 pandemic have evaluated psychological stress or the impact of the pandemic on dental practice. There are no studies in the literature, that have evaluated various home and workplace factors affecting the overall work-life balance in a dental professional’s life during the COVID-19 pandemic lockdown. Hence it indispensable to explore these factors.

Specifically, in the light of the pandemic, our study has focussed on multiple factors like relationships, workplace dynamics, activities, preventive measures, mental health, and physical health. We have adopted the structural equation modelling (SEM) approach and PLS-SEM (Partial least Square) which enables researchers to examine complex models with large variables [14]. The present study was conducted to evaluate the work-life balance amongst dental professionals during COVID-19 lockdown. The objective of the study was to understand the changes they underwent both at home and professional front with a hypothesis that physical and mental health, activities, relationship status, and workplace influence the work-life balance significantly. The finding of our study will facilitate to identify the weaker links and strategise with newer functional policies, better working culture, enhanced readiness to face newer challenges and protect the well-being of oneself, and sustain the workforce during every other emergency or pandemics in the future

## Materials and methods

A cross-sectional online survey was conducted after the approval from the Institutional Ethics Committee (IEC- 392/2020). A self-administered questionnaire was designed on Google Forms. The questionnaire was framed based on information from the previous pandemic, COVID-19, and its effects. It was pilot tested for feasibility and interpretation. The interpretations were validated with twenty participants of the same survey group. Based on the feedback the final questionnaire was prepared. It was distributed through e-mail, WhatsApp, and social media platforms (Facebook, Twitter) to reach out to as many dentists as possible. The participation was voluntary and participants had to consent for the same by selecting the consent form before answering the questionnaire. It was conducted for 2 months (April 2020 to June 2020) during the COVID-19 pandemic lockdown. Dental professionals of various specialties working in private clinics and/or institutional attached hospitals, with age 25 years and above, who consented to participate in the study were included. The sample size was calculated based on the structural equation model analysis planned for the study. This analysis requires the N:q ratio to be 5 to 1 or 5 observations (participants) for each estimated parameter in the model. Hence a minimum of 175 responses was required for our study.

The survey tool consisted of five major sections, the first section gathered demographic details along with their qualification, experience, and workplace details. The second section was designed to determine changes in their activities during the lockdown period. The third section of the survey assessed their relationship changes during the lockdown. The fourth section consisted of questions about their approach towards physical health like modifications and engagement in physical activities, pre-and post-lockdown, and mental health-related questions.

The fifth section evaluated their details regarding work status and preventive measures followed, and also assessed their awareness about COVID-19 prevention.

### Statistical analysis

The response gathered was collected in an excel sheet and was analyzed using Smart PLS® version 3.0.

Descriptive statistics was performed on the demographic data using IBM SPSS Version 20. Structural equation modelling (SEM) was chosen to evaluate the interaction of various factors like activities, physical and mental health, relationship status, and workplace on the work-life balance of dental professionals. To assess the factors affecting work-life balance, the collected data were analyzed in three steps. In the first step, factor analysis was applied to the statements asked the respondents for collecting data on dimensions of influencing factors and affecting the work-life balance. Further, these variables were confirmed through factor loadings. In the second step, SEM was applied with the help of Smart PLS software showing the impact of influencing factors on work-life balance. In the third step, the results obtained through SEM-PLS and t-statistics were reported and interpreted.

## Results

Out of 500 dentists approached with the questionnaire, 180 responses were obtained (response rate 37.5%) with 127 female dentists and 57 male dentists. The majority of the participants belonged to the age group of 25–30 years (50.3%), 31–40 years (35.8%), 21–50 years (12.8%). 62% (115) of the dentists were specialists with 56.6% (104) with an MDS degree and 3.3% (5) with a Ph.D. degree. Most of the participants 53.3%(n = 96) were married and 89.4%(n = 161) of them were in a full-time job. The demographic and workplace details are described in Table 1.

**Table 1:** Demographic and workplace details.

Variable	Responses	Frequency	Percentage
**Gender**	Female	127	70.6
	Male	53	29.4
**Age**	25–30 Years	93	50.3
	31–40 Years	67	35.8
	41–50 Years	18	12.3
	50–60 Years	2	1.6
**Specialty**	General Dentist	65	38
	Specialist	115	62
**Highest qualification**	BDS	71	40.1
	MDS	104	56.6
	PhD	5	3.3
**Years of experience**	0–5 yrs	97	54
	6–10 yrs	38	21
	11-15yrs	20	11
	More than 15	25	14
**Where do you practice?**	Dental College	83	46.1
	Free-Lancing	14	7.8
	Government Hospital	5	2.8
	Group Practice	13	7.2
	Mixed	19	10.6
	Private Hospital	46	25.6
**Time dedicated to your job**	Full Time	161	89.4
	Part-Time	19	10.6
**Marital status**	Divorced	2	1.1
	Married	96	53.3
	Single/ Unmarried	82	45.6
**Do have any children?**	No	108	60
	Yes	72	40
**Your usual living status**	Joint family	41	22.8
	Living alone	30	16.7
	Nuclear family	109	60.6
**Total**		**180**	**100**

Results of the descriptive analysis of changes in physical activity, physical and mental health, and the relationship status is depicted in Table 2. The majority of the participants (65%) had inculcated a new hobby during the lockdown period. The duration of involvement in physical activity during lockdown increased compared to before the lockdown. Around 57.2%(n = 103) of dentists responded to have altered sleep and 25.6%(n = 46) expressed to be in an anxious state during the lockdown. 36.1% (n = 65) of participants admitted to having changes in their mental health more than physical health, where as28.3%(n = 51) responded to have no changes in both. However, 21.7% (n = 39) reported only physical health to be affected. 38.3%(n = 69) of participants reported to have changes in relationship status with their family members and 23.9% (n = 43) of them reported improvement in relationship status with their spouse.

**Table 2:** Effect of Lockdown on their regular activities, physical and mental health, and relationship.

**Have you inculcated any new hobby or habit?**	No	63	35
	Yes	117	65
**Screen time per day before lockdown**	<1 hour	19	10.6
	1–2 hours	68	37.8
	2–4 hours	56	31.1
	4–6 hours	24	13.3
	>6 hours	13	7.2
**Physical activity before lockdown**	Nil	25	13.9
	<1 hour	95	52.8
	1–2 hours	53	29.4
	2–3 hours	5	2.8
	>3 hours	2	1.1
**Physical activity during lockdown**	Nil	8	4.4
	<1 hour	99	55
	1–2 hours	60	33.3
	2–3 hours	9	5
	>3 hours	4	2.2
**Overall how do you feel days are passing during the lockdown**	Becoming lazy and lethargic	86	47.8
	More active	33	18.3
	Same	61	33.9
**Mood changes during the lockdown?**	Anxious	46	25.6
	Happy	32	17.8
	Irritable	36	20
	Sad	8	4.4
	Same as earlier	58	32.2
**Changes in sleep cycle during lockdown**	Altered	103	57.2
	Remained same	77	42.8
**Changes in the mental and physical health status during lockdown**	Only physical health affected	39	21.7
	Physical health more than mental	6	3.3
	No change in physical and mental health	51	28.3
	Mental health more than physical	65	36.1
	Only mental health affected	19	10.6
**Change in relationship with family members after lockdown**	Yes	69	38.3
	No	79	43.9
	May be	32	17.8
**Change in relationship with the spouse**	Feels strained	14	7.8
	Improved	43	23.9
	Remains same	40	22.2

When questioned about their work and workplace, a striking observation was that a majority of dentists felt like resuming work 83.4%(n = 156), 91.4% (n = 171) reported adequate availability of PPE at their workplace. 43.4% (n = 81) also felt safe going to the workplace. Most of the dentists were practicing teledentistry 51.9%(n = 97) and about 76.5% (n = 143) were involved in spreading awareness about the pandemic. Detailed description regarding their workplace details is given in Table 3.

**Table 3:** Details of the workplace and preventive measure for COVID-19.

**How many days have you visited the workplace during the lockdown?**	0	62	33.2
	1–5 days	35	18.71
	6–10 days	16	8.55
	11–15 days	19	10.16
	More than 15	55	29.41
**Do you feel like resuming your work?**	No	31	16.5
	Yes	156	83.4
**Are you practicing Teledentistry?**	No	97	51.9
	Yes	90	48.1
**Do you feel safe going to the Workplace in this situation**	Yes with adequate precautions	81	43.4
	No	61	32.6
	Not sure	45	24
**Does your workspace provide Protective material**	Yes	171	91.4
	No	16	8.6
**Have you taken part in spreading awareness about COVID-19**	Yes	143	23.5
	No	44	76.5

Table 4 demonstrates work-life balance, as calculated from a factor analysis of the relevant indicators. It shows the demographic profile of the respondents, specifically their gender, marital status, place of practice, and, their usual living status significantly influenced the work-life balance. However, work-life balance is independent of age and qualification.

**Table 4:** Illustrates the model fit of the regression between work-life balance and the demographic variables.

Source	Type III Sum of Squares	df	Mean Square	F	Sig.
**Model**	65.256a	53	1.231	1.375	.076
**Gender**	4.385	1	4.385	4.896	.029
**Marital Status**	8.754	3	2.918	3.258	.024
**Age group**	4.616	4	1.154	1.289	.278*
**Highest Qualification**	36.171	36	1.005	1.122	.314*
**Place of Practice**	12.133	6	2.022	2.258	.042
**Usual Living Status**	3.759	2	1.879	2.098	.127
**Error**	113.744	127	.896		
**Total**	179.000	180			

a. R Squared = .365 (Adjusted R Squared = .099)

* not significantly influencing

### Measurement model

The first step before estimating the structural model is to evaluate and refine the measurement model. For this, the outer loadings of all the pertinent items in the survey were obtained and statistically tested for significance. Indicators with statistically insignificant outer loadings were dropped from the analysis as these would not correlate well with the underlying constructs. The AIC (Akaike Information Criterion) BIC (Bayesian Information Criterion) and HQC (Hannan- Quinn Information Criterion) values output by Smart-PLS (version 3.3) were also monitored while refining the model. According to Hair et. al. (2019), outer loadings above 0.708 are recommended because they account for more than 50 percent of the indicator’s variance. Almost all the outer loadings of the selected items are above 0.708 [14].

Further, to test the construct reliability and validity, the usual measures of internal consistency were calculated. Table 5 displays the obtained composite reliability measures of the dimensions based on a bootstrapping procedure with 2000 samples. The composite reliability values range between 0.6 and 0.9 for most of the constructs indicating acceptable internal consistency reliability [14]. The small p-values indicate the significance of the measures at the 5% level.

**Table 5:** Composite reliability measures of constructs, Bootstrapping with 2000 samples.

	Original Sample (O)	Sample Mean (M)	Standard Deviation (STDEV)	T Statistics (|O/STDEV|)	P Values
**Activities**	0.903	0.860	0.129	6.977	0.000
**Mental Health**	0.393	0.327	0.137	2.868	0.004
**Physical Health**	0.351	0.312	0.175	2.004	0.045
**Preventive Measures**	0.636	0.573	0.113	5.604	0.000
**Relationships**	0.699	0.695	0.042	16.827	0.000
**Work Life Balance**	0.540	0.541	0.073	7.434	0.000
**Work Place**	0.318	0.269	0.162	1.967	0.049

To assess the convergent validity, the average variance values (AVE) were calculated as shown in Table 6. The AVE values are all-around 0.5, and significant as observed from the small p-values [14]. Thus, convergent validity is established. The AVE values are all-around 0.5, and significant as observed from the small p-values [14]. Thus, convergent validity is established.

**Table 6:** AVE values of constructs, Bootstrapping with 2000 samples.

	Original Sample (O)	Sample Mean (M)	Standard Deviation (STDEV)	T Statistics (|O/STDEV|)	P Values
**Activities**	0.612	0.563	0.113	5.410	0.000
**Mental Health**	0.238	0.228	0.036	6.620	0.000
**Physical Health**	0.378	0.367	0.033	11.495	0.000
**Preventive Measures**	0.378	0.372	0.034	10.965	0.000
**Relationships**	0.499	0.501	0.029	17.345	0.000
**Work Life Balance**	0.469	0.475	0.033	14.173	0.000
**Work Place**	0.399	0.385	0.049	8.115	0.000

To test the discriminant validity of the constructs, the HTMT (Heterotrait and Monotrait)ratios were calculated as given in Table 7. Almost all the ratios, are less than 0.85, indicating that the constructs are all distinct from each other [14].

**Table 7:** HTMT ratios of constructs.

	Activities	Mental Health	Physical Health	Preventive Measures	Relationships
**Mental Health**	0.249				
**Physical Health**	0.284	1.032			
**Preventive Measures**	0.219	0.544	1.029		
**Relationships**	0.138	0.531	0.703	0.476	
**Work Life Balance**	0.258	0.580	0.602	0.642	1.117
**Work Place**	0.267	0.668	0.491	0.764	0.349

### Structural model

Before Structural Equation Modelling, the collinearity among the constructs was examined and the Variance Inflation Factor (VIF) values are given in Table 8. It shows that all the VIF values are less than 3, indicating the absence of multicollinearity among constructs. Hence, there will not be any bias in the regression results [14].

**Table 8:** VIF values of constructs.

	Work-Life Balance
**Activities**	1.017
**Mental Health**	1.190
**Physical Health**	1.068
**Preventive Measures**	1.035
**Relationships**	1.118
**Work Place**	1.040

**Fig 1:**
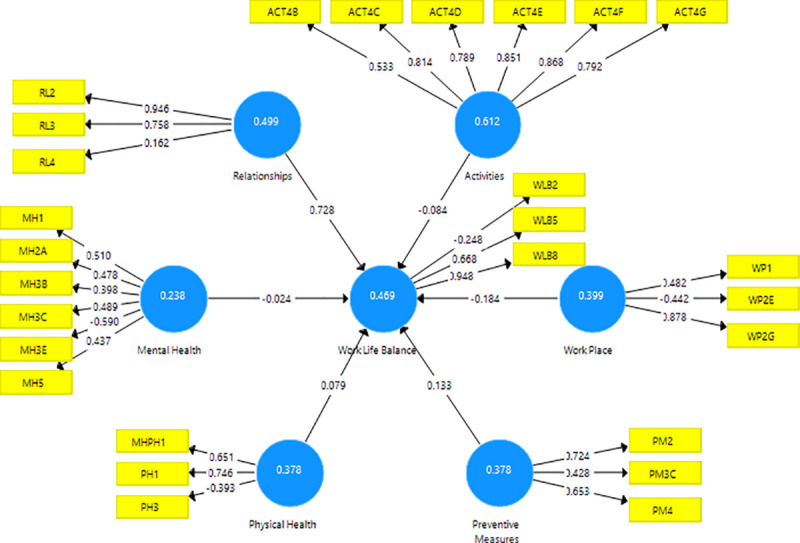
Path coefficients and outer loadings for the direct relationship between the influencing factors and work-life balance.

Fig 1 shows the path coefficients and outer loadings for the direct relationship between the influencing factors and work-life balance, such as relationships, mental health, workplace, and physical health. R^2^ value is a measure of the explanatory power of the structural model, which indicates the proportion of variation in the dependent construct that is explained by the independent constructs. With this value being equal to 0.627, the strength of the relationship is said to be moderate to quite substantial [14].

**Fig 2:**
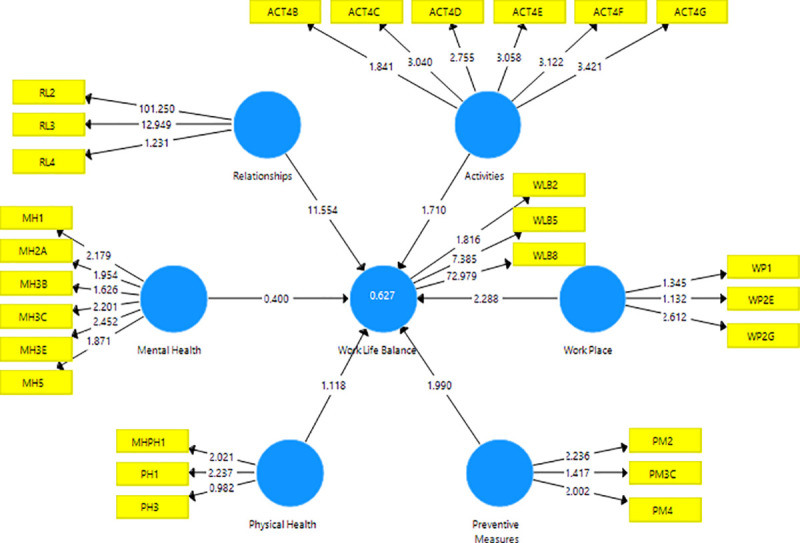
t-statistics for the inner and outer models.

Fig 2 depicts the estimated PLS-SEM with corresponding t-statistics for each of the indicators and constructs in the model. The t-statistics are used to check the significance of the path coefficients as well as indicators (Fig 2). p-values, corresponding to these t-statistics, are less than 0.1 (0.05), then the corresponding effects are significant at the 10% (5%) level of significance.

Table 9 gives the total effect values for each of the constructs. From Table 9, it can be inferred that relationships, particularly those with parents/children/siblings and spouse, have a strong positive impact on work-life balance. Workplace factors, like wearing gowns and N95 face masks and the number of days one visited the workplace during the lockdown, negatively influence work-life balance (2.5% level of significance). Preventive awareness measures, like cross-checking the authenticity of the messages received on social networks, following hand hygiene routinely at home during the lockdown, and making COVID-19 awareness-related phone calls, are positive significant contributing factors influencing work-life balance (5% level of significance). Activities, like browsing social networks, watching news/videos/movies, mobile/video games, and research work, are marginally significant negative influences on work-life balance (10% level of significance). However, no significant evidence to support the influence of physical health factors, like energy level, sleep duration, and overall health status on work-life balance was seen.

**Table 9:** Path coefficients, t-statistics, and p-values of the structural model.

	Original Sample (O)	Sample Mean (M)	Standard Deviation (STDEV)	t-statistics (|O/STDEV|)	p-values
**Activities -> Work Life Balance**	-0.084	-0.093	0.049	1.710	0.087
**Mental Health -> Work Life Balance**	-0.024	0.015	0.059	0.400	0.689
**Physical Health -> Work Life Balance**	0.079	0.075	0.071	1.118	0.264
**Preventive Measures -> Work Life Balance**	0.133	0.125	0.067	1.990	0.047
**Relationships -> Work Life Balance**	0.728	0.706	0.063	11.554	0.000
**Work Place -> Work Life Balance**	-0.184	-0.158	0.080	2.288	0.022

t-test statistics > 1.96 or p-value < 0.05 is considered as statistical significant.

## Discussion

Dental professionals are often conflicted with playing the dual roles; at the professional front and home. Studies performed on health care workers, during the SARS outbreak in 2002 revealed the clash between ‘selflessness and professional liability with ‘fear of risking their family and relatives, resulting in remarkable mental and physical burden [5, 15]. About 18–57% of healthcare workers exhibited fear of infection, concern for family, job-related stress, and attachment insecurity during the SARS outbreak [5, 9, 10]. Increased mental stress and physical burden can disrupt the work-life balance, especially when the ‘work’ is demanding. It can adversely influence their decision-making ability and efficiency leading to suboptimal patient care and productivity both at home and at the office [16]. In the present study, we have evaluated various factors in a dentist’s daily life at home and at the workplace, which have altered during the COVID-19 lockdown period.

The current study showed a lower response rate, which was comparable to some of the previous studies [4, 5, 17]. This can be attributed to the restricted timeline of conducting the survey, as we intended to capture the effects during the lockdown, to avoid the fading off of the effect of variables with time. The majority of the participants were females and young professionals, this was in consensus to the earlier [17, 18]. Marital status and number of children exhibited a significant impact on work-life balance as revealed by factor analysis.

Path models also showed mental health, physical health, activities, relationship, and workplace parameters to influence the work-life balance. A study by Shacham M et. al. (2020) evaluated factors causing psychological distress among dentists in Israel, and results revealed gender and relationship status to have effects on psychological distress [5]. Another study by Mijiritsky E et al. (2020) assessed the association between ‘subjective overload’ like aspects from everyday life other than dental practice, and psychological stress among dentists. They determined subjective overload to have a strong influence on psychological stress [19]. A study on Chinese medical staff during COVID-19 lockdown reported that constant contact with their respective families through video calls led to reduced psychological stress with boosted morale and improved mental health [20]. The SEM analysis performed also showed that relationships with parents, siblings, children, and spouse positively affect the work-life balance. Age is also an important factor in stress and stress reactions [21, 22]. As the age advances, dentists acquire financial security and thus have limited stress as opposed to their younger counterparts, who are in the process of financially establishing themselves. The financial uncertainty that the present COVID-19 brings could be a foremost reason for stress among dentists [3].

During the lockdown, maximum participants in our study were involved in academic work, indoor game activities, and spending quality time with family. More than half of the respondents had inculcated new hobbies and improved relationships with their spouse during the lockdown. Even though these results cannot be compared with the previous studies as similar patterns have not been evaluated earlier. The improved relationship status can be due to the more quality time spent with family during the lockdown period.

In the present study, it is observed that many of the dentists felt helpless and anxious. This has affected either their mental or physical health with a majority having effects on both. Similar results were noted by Ugo Consolo et al. (2020) on Italian dentists, where 4.2% experienced fear intensely, 6.2% reported experiencing anxiety, 12.6% of respondents felt intensely sad [4]. Shacham et. al. (2020) also reported similar concerns [5]. The majority of the respondents reported changed/altered sleep patterns. This could be attributed to the fear, anxiety, and uncertainty of life during the pandemic. Altered sleep pattern has been associated with an increased medical error rate, interpersonal conflicts, and reduced peak performance [23].

The majority of female dentists felt unsafe resuming the work post lockdown, in comparison to male dentists. They also reported irritability, anxiety, and changes in their sleep pattern affecting their mental and physical health. Similar studies on women endodontists and a large population revealed overall higher stress level in females during the COVID-19 pandemic [3, 24, 25]. Previous pandemics such as SARS, Swine flu, and Bird flu also reported of negative impact on women which lasted even post-pandemic and the same results are being observed in the current pandemic. It is highly crucial to make practical policies and interventions to have a sustainable social and economic environment to address the work-life balance issues for women [24].

The participants stated adequate availability of Personal Protective Equipment and also showed a positive attitude towards resuming work post lockdown. This constructive approach seen towards re-joining work can be related to the fact that they were associated with an academic and clinical setup. During this pandemic, teledentistry and telemedicine have gained more focus and usage. It has helped in giving consultation, pre-operative evaluation, and postoperative evaluation of patients without patients having to come to clinics thus reducing exposure to the infection [4, 17]. In the present study, half of the total respondents reported using teledentistry during lockdown for various purposes. A few of the dentists in our study reported working even during the lockdown period as a part of emergency healthcare service providers. However, these findings were in contrast with previous studies by Ugo Consolo et al. (2020) Shacham et. al. (2020) [4, 5].

The results of the present study reflected a discrepancy in the work-life balance of dentists during the lockdown which showed signs of unpreparedness. A strengths, weakness, opportunities, and threats (SWOT) analysis helps policymakers to provide sound and strategic planning for handling emergency outbreaks in the future. Hospital administrators and dental clinic owners should prioritize their preparedness plans in terms of financial consequences, and attrition of the workforce during such pandemic [6, 26].

Self-reporting bias and confounding factors like non-consideration of dentists from all Indian states and low response rates are the limitations of our study. Further studies, in this direction, have to explore the role of other possible confounders like considering the standard scales for stress assessment and association with work and other factors through model analysis [3]. A comparative study, with the effects of pandemics during the lockdown and unlock period, is also required to analyze pre and post effects of work-life balance on dental professionals.

## Conclusion

Our study supported the hypothesis that factors like physical and mental health, activities, relationship status, and workplace influence the work-life balance. A significant change in the work-life balance during the COVID-19 pandemic was seen amongst female dental professionals. Hence, there is a need to make professional mental health services easily accessible to all dentists during such unforeseeable future emergencies. As an evolutionary phase where new advances are expected to evolve, dentists should emerge out successfully from this crisis. Focus on exit strategies for dentists by formulating better working protocols for sterilization/disinfection, cost-effective, faster, reliable screening and diagnostic tools, making barrier materials available, and a robust mental health support system is essential.
